# *BJS* commission on the surgical management of the axilla in breast cancer

**DOI:** 10.1093/bjs/znaf125

**Published:** 2025-09-16

**Authors:** Michael R Boland, Oreste D Gentilini, G Bruce Mann, Jana de Boniface, Elena Leinert, Christine Obondo, Thorsten Kühn, Anna Weiss, Judy C Boughey, Paul T R Thiruchelvam, Anna Duncan, Meera Joshi, Maria Mani, Isabel T Rubio, Pedro F Gouveia, Malin Sund

**Affiliations:** Department of Breast Surgery, St Vincent’s University Hospital, Dublin, Ireland; Università Vita-Salute San Raffaele, Milan, Italy; Breast Surgery Unit, IRCCS Ospedale San Raffaele, Milan, Italy; Breast Service, The Royal Melbourne Hospital, Parkville, Victoria, Australia; Department of Surgery, The University of Melbourne, Parkville, Victorria, Australia; Department of Surgery, Capio Saint Göran’s Hospital, Stockholm, Sweden; Department of Medical Epidemiology and Biostatistics, Karolinska Institutet, Stockholm, Sweden; Department of Gynaecology and Obstetrics, University of Ulm, Ulm, Germany; Department of Medical Epidemiology and Biostatistics, Karolinska Institutet, Stockholm, Sweden; Department of Surgery, South General Hospital, Stockholm, Sweden; Department of Gynaecology and Obstetrics, University of Ulm, Ulm, Germany; Breast Centre, Die Filderklinik, Filderstadt, Germany; Division of Surgical Oncology, Department of Surgery, University of Rochester, Rochester, New York, USA; Wilmot Cancer Institute, University of Rochester, Rochester, New York, USA; Division of Breast and Melanoma Surgical Oncology, Department of Surgery, Mayo Clinic, Rochester, Minnesota, USA; Department of Cancer & Surgery, Imperial College, London, UK; Department of Plastic Surgery, University of Upsala, Upsala, Sweden; Department of Cancer & Surgery, Imperial College, London, UK; Department of Plastic Surgery, University of Upsala, Upsala, Sweden; Clinica Universidad de Navarra, Centro de Cáncer Clínica Universidad de Navarra, Madrid, Spain; Department of Surgery, Universidad de Navarra, Pamplona, Spain; Breast Unit, Champalimaud Foundation, Lisbon, Portugal; Lisbon School of Medicine, University of Lisbon, Lisbon, Portugal; Department of Diagnostics and Intervention/ Surgery, Umeå University, Umeå, Sweden; Department of Surgery, University of Helsinki and Helsinki University Hospital, Helsinki, Finland

## Introduction

The management of breast cancer has evolved dramatically over the past 25 years. In stark contrast to the radical mastectomy proposed by Halsted^1^ over 100 years ago, patients can benefit from individualized treatment programmes with less extensive surgery, possibly with oncoplastic techniques, complex and highly effective chemotherapeutic regimens (±immunotherapy and human epidermal growth factor receptor 2 (HER2) direct therapy), and focused radiation. Surgery has become more refined and offers the maximal benefit, whilst minimising morbidity. It is the surgical management of regional axillary lymph nodes that however has undergone the most significant changes over the last 25 years. Whilst all patients were previously subjected to an axillary lymph node dissection (ALND), this morbid procedure is now reserved for those with advanced lymph node involvement. The therapeutic value of axillary surgery is limited to specific cases and such surgery is now performed predominantly for staging and prognostic purposes as well as guiding, in selected cases, the provision of adjuvant therapy. Surgeons continue to investigate whether a more minimal approach to the axilla in patients with breast cancer could be employed. Conversely, there remains a group of patients who still require ALND and techniques to minimise associated complications, such as lymphoedema, have been increasingly investigated. As the rate of change within this area continues to increase, the purpose of this BJS Commission is to provide an overview, at this point, of all aspects of surgical management of the axilla including future considerations.

To undertake this overview, the editorial board of the BJS selected experts within the different domains of axillary management of breast cancer. All those selected had a significant record of high-quality publications within their special interest. Once a list of expert authors was compiled, it was then approved by the BJS Editorial team and of those invited, all agreed to partake within this Commission. All authors were also asked to include ‘future experts’ at a senior trainee or early consultant level, to complement the authorship group. Authors were then split into five groups, each tasked with providing a detailed contemporary review of certain key aspects of axillary surgery. By selecting an international group of experts, the aim was to formulate a balanced and collaborative analysis that represents practice within this realm of breast surgery, globally. The overview was approved by the BJS Editors.

.

### Part 1—De-escalation in early-stage breast cancer patients undergoing primary surgery: omission of sentinel lymph node (SLN) surgery and its implications for adjuvant treatments

Oreste D. Gentilini and G. Bruce Mann

#### Background

Before mammographic screening, most patients diagnosed with breast cancer had lymph nodes involved at the time of surgery, and the value of nodal dissection to achieve local control as well as determine prognosis and guide adjuvant therapy was generally accepted. In settings with established population-based mammographic screening ∼65% have no nodal involvement and the role of nodal surgery has been reassessed^2^. Major improvements in breast cancer outcomes have been attributed to a combination of early diagnosis due to mammographic screening and the introduction, refinement, and widespread use of adjuvant systemic therapies^3^. A nationwide study from the Netherlands compared cohorts from 1999–2005 and 2006–2012. The latter cohort was more likely to have T1 cancer (65% *versus* 60%) and be node negative (68% *versus* 65%) but received more chemotherapy and endocrine therapy. The relative 5-year survival of those with T1 cancer was close to 100%, and that of all node-negative patients was 95% in the earlier cohort and 98% in the latter^4^. The large numbers of patients being diagnosed at a stage when outcomes are excellent offers the potential for safe de-escalation of therapy. De-escalation of breast surgery was a feature from 1980 to 2000^5^, and since then a focus of trials has been on de-escalation of axillary surgery^6–9^ and adjuvant radiotherapy^10^ for those with early breast cancer.

#### Evolution of surgical staging of the axilla

Sentinel lymoh node (SNL) surgery was introduced in the 1990s after it was demonstrated that lymphatic drainage is predictable and reproducible, and that the false negative rate (FNR) of SLN surgery was under 10% with minimal apparent clinical impact of these false negatives^6,7^. Once the safety of SLN surgery in pN0 breast cancer was established, the need for completion axillary dissection in those with low volume disease in the sentinel node was questioned^8,11,12^. Later in this commission Professors de Boniface and Kuehn outline developments and the status of axillary treatment in those with a positive SLN. While SLN surgery has substantially lower morbidity than axillary dissection, it remains potentially morbid^13^. Improvement in preoperative assessment of the axilla via focused ultrasonography has meant there is less unexpected nodal involvement, and the extent of such involvement is less than in the past^14^. In addition, systemic therapy recommendations are more dependent on biological features of the index cancer, diminishing the importance of the results of axillary staging^15^. Due to these considerations, trials have been designed to evaluate the safety of omitting all axillary surgery. SOUND^16^ and INSEMA^17^ have been recently published while BOOG 13-08^18^ and NAUTILUS^19^ have not been released yet. The SOUND trial was an international RCT of 1463 patients with the primary endpoint being distant disease-free survival at 5 years. Patients of any age with cT1 N0 early breast cancer and non-suspicious axillary ultrasound were randomized to receive either SLN surgery or no axillary surgery. The SOUND trial reported the non-inferiority of omitting axillary surgery, with an extremely low incidence of breast cancer-related events in both of the study arms: axillary relapse was <1%, ipsilateral breast cancer recurrence was 1%, distant metastases was 2%, and contralateral breast cancer was diagnosed in 1% of patients. Adjuvant treatment recommendations did not differ between the two study arms suggesting that the information regarding nodal status did not substantially affect multidisciplinary team recommendations. The INSEMA trial has similar findings to SOUND, randomizing patients with clinically node-negative T1 or T2 cancer to treatment with or without SLN surgery. There were no differences in local or regional recurrence and no survival differences. Importantly, most patients were postmenopausal, clinically and pathologically stage 1, oestrogen receptor (ER) positive, and HER2 negative; 95% received adjuvant endocrine therapy, 12% received adjuvant chemotherapy, and patients not receiving postoperative radiotherapy were excluded. SOUND and INSEMA provide very strong evidence that patients with clinical stage 1 cancer and a negative axillary ultrasound can safely omit axillary surgery, so long as the lack of pathological information does not affect the postoperative treatment plan. Results of longer-term follow-up of these trials will be important to confirm that the findings persist after the cessation of adjuvant endocrine therapy.

#### Significance of axillary nodal status

Whether pathological confirmation of nodal status matters will depend on local practice patterns. The MIRROR study of 2707 patients assessed the prognostic significance of micrometastases or isolated tumour cells (ITCs) as well as the impact of adjuvant systemic therapy^20^. Groups with negative nodes and those with ITCs or micrometastases were identified, as were those in the latter group that did or did not receive adjuvant systemic therapy. There was a sizeable difference in 5-year disease-free survival between those with and without nodal disease (76.5% *versus* 85.7%, *P* < 0.001) in those that did not receive adjuvant systemic therapy. In those with nodal disease, receipt of adjuvant therapy was associated with a substantial improvement in 5-year disease-free survival (76.5% *versus* 86.2%, *P* < 0.001).

In some groups, nodal status may impact the nature of systemic therapy recommendations in those with cT1 N0 early breast cancer. The RxPONDER trial suggests that chemotherapy may be of benefit to all those aged under 50 years with nodal involvement, irrespective of results of genomic expression profiling^21^. In addition, the recommended duration of adjuvant endocrine therapy may vary with the risk estimate and is usually extended beyond 5 years in patients with node-positive disease. The absence of nodal involvement might allow de-escalation of the hormonal treatment, both in terms of drug choice (tamoxifen *versus* aromatase inhibitors or ovarian suppression *versus* no ovarian suppression) and duration, especially in the case of substantial adverse effects. Published evidence from two large centres in the USA describes the adoption of criteria (patients either postmenopausal or aged 60–69 years, grade 1–2, ER positive, presence of invasive ductal carcinoma, and absence of lymphovascular invasion) similar to those utilized in the SOUND trial with good effect^22,23^. Recently published American Society of Clinical Oncology guidelines^24^ have been updated to incorporate the results of the SOUND and INSEMA trials.

#### Optimization of other modalities

Over recent decades there has been progressive de-escalation of surgical therapy with impressive results as evidenced by this *BJS* Comission on axillary surgery. Notably, in trials of de-escalation of surgical treatment, standard therapy with radiotherapy after breast-conserving surgery (BCS) and adjuvant systemic therapy has been used in almost all patients. A patient perspective when considering breast cancer treatment is vital. McIntosh *et al*.^25^ asked the question of which treatment patients would most like to omit. Chemotherapy, endocrine therapy, surgery, and radiation were nominated by 36%, 31%, 21%, and 10% of participants respectively. This implies that all treatment modalities should be considered when pursuing the aim of treatment optimization. Trials investigating radiotherapy omission based on traditional low-risk pathological findings have found that radiotherapy omission is associated with a local recurrence rate at 10 years of around 10%, compared with around 2% with radiotherapy, but no difference in breast cancer-specific survival^10,26,27^. Ongoing trials are investigating breast cancer subtypes to identify a low-risk group. The LUMINA, PRIMETIME, PRECISION, and EXPERT trials are investigating the use of the luminal A phenotype, IHC4, and PAM50, respectively, while IDEA and DEBRA are using OncotypeDX to identify a low-risk group for whom radiotherapy may be safely omitted after BCS. The BR-008 HERO study is assessing radiotherapy omission in favourable HER2 positive cancers. The PROSPECT trial represents an alternative approach to de-escalation of treatment^28^. PROSPECT used MRI findings and pathology to identify patients in whom radiotherapy may be safely omitted. The hypothesis was that many local recurrences seen after BCS alone in stage 1 breast cancer are due to areas of malignant disease not identified on routine imaging but identifiable on MRI. Of 443 clinically stage 1 non-triple-negative breast cancer (TNBC) patients, 48 (11%) had biopsy-proven additional areas of early cancer or ductal carcinoma *in situ*, all of whom were treated at the time of surgery for the index lesion; 201 of 443 were eligible for radiotherapy omission in PROSPECT and the ipsilateral local recurrence rate at a median follow-up of 5 years was 1%. Intriguingly, there were no distant metastases from the index cancer at the median follow-up in the entire cohort. The EUROPA trial is assessing whether partial breast radiotherapy can be used instead of endocrine therapy in women aged >70 years with luminal A-like early-stage breast cancer^29^. A preplanned interim analysis has shown less impact on health-related quality of life with radiotherapy compared with endocrine therapy although recurrence outcomes are not yet available^30^. Ninety percent of patients were pathologically node negative, with nodal status unknown in 9%. Efforts to find patients for omission of radiotherapy or endocrine therapy are focused on node-negative patients. Caution is appropriate when applying results from trials of de-escalation of adjuvant therapies in patients with pathologically negative sentinel nodes to those with uncertain nodal status given the importance of this disease demonstrated in the MIRROR study^20^.

#### Summary

SLN surgery has been a standard recommendation for most patients with invasive breast cancer since it replaced axillary dissection as a less invasive method of axillary staging over 25 years ago. Evidence has accumulated that SLN surgery is not necessary in patients receiving modern multimodality treatment. The Choosing Wisely guidelines of the Society of Surgical Oncology recommend omission of SLN surgery in patients older than 70 years with small ER-positive HER2-negative breast cancer when the adjuvant treatment plan is clear and does not include the addition of chemotherapy to endocrine treatment. The SOUND and INSEMA trials support these recommendations and suggest that women aged over 50 years can reasonably avoid sentinel node biopsy so long as the results of SLN surgery would not affect the postoperative treatment plan. Optimizing treatment of low-risk early-stage breast cancer will require coordinated optimization of surgery, radiation, and systemic therapy. It will be important to ensure that advances in de-escalation of one aspect of treatment do not preclude other improvements. Patients must be included in these decisions to ensure that their individual perspectives are taken into account.

### Part 2—De-escalation of upfront axillary surgery in patients with sentinel node-positive breast cancer

Jana de Boniface, Elena Leinert, Christine Obondo and Thorsten Kühn

#### Background

ALND was the only surgical staging method in breast cancer until the introduction of SLN surgery at the beginning of this century. Advances in early detection, population-based screening, and increased awareness have led to a significant downwards shift in staging, making ALND less appropriate in most settings due to the high risk of long-term arm morbidity. A recent meta-analysis compared the prevalence of arm-related complications between ALND and SLN surgery alone: arm lymphoedema after >2 years, 23.6% *versus* 5.9%; pain, 32.9% *versus* 21.7%; limited range of motion, 29.8% *versus* 17.1%; and reduced strength, 30.9% *versus* 15.2%^31^. Given these concerns, there has been interest in less invasive approaches to managing the axilla in patients with limited SLN involvement. These strategies aim to minimize arm morbidity without compromising survival outcomes, especially as modern treatment strategies have improved disease control and shifted the focus towards survivorship. This section examines the current role of ALND and explores further de-escalation options for patients with breast cancer and limited axillary involvement.

Several prospective randomized trials have shown the non-inferiority of the omission of completion ALND in patients with clinically node-negative breast cancer and one to two SLN(s) with metastases who undergo primary surgery^11,12,32–35^. Radiotherapy schedules, however, varied (*Table 1*). Hence, international guidelines do not recommend completion ALND in patients with limited axillary involvement, while disparity remains on recommendations for adjuvant radiotherapy. An interesting difference between these trials is the use of preoperative axillary ultrasound (AUS): in the ACOSOG Z0011 and AMAROS trials, AUS was optional, while the SINODAR-ONE and OTOASOR trials required a negative AUS for eligibility. SENOMAC mandated AUS but allowed enrolment of patients with suspicious, non-palpable lymph nodes in whom fine-needle aspiration cytology or core biopsy confirmed axillary metastases. AUS assessment of the axilla has limitations due to its user dependency and dynamic nature, which impact reproducibility. The Breast Imaging Reporting and Data System (BI-RADS) lacks strict criteria for the ultrasound assessment of the axilla. Features such as cortical or eccentric cortical hypertrophy ≥3 mm, a round shape, or the loss of central hilum typically indicate suspicious nodes^36,37^. Moreover, AUS sensitivity and specificity vary significantly (26.4% to 75.9% and 88.4% to 98.1% respectively)^38^. Integrating deep-learning radiomics with combined imaging and clinical criteria may improve AUS performance and enhance its role in clinical decision-making in the future^39^.

**Table 1:** Prospective randomized trials on the omission of completion ALND in patients with clinically node-negative breast cancer

Trial	Study population	Type of breast surgery	Cohort	RT	Results
Z0011	cT1–T2 cN0 pN1(sn) 1–2 macrometastases and micrometastases	BCS 100%	SLNB alone *versus* ALND	Tangential WBRT Supraclavicular fossa RT 15%, high tangents ≥50%, nodal RT 19%	10-year results Overall survival: 86.3% (SLNB) *versus* 83.6% (ALND) Disease-free survival: 80.2% (SLNB) *versus* 78.2% (ALND) Axillary recurrence rate: <1% (SLNB) *versus* 0 cases (ALND)
AMAROS	cT1–T2 cN0 pN1(sn) unrestricted number of metastases (95% 1–2 metastases)	BCS 82% Mastectomy 18%	ART *versus* ALND	WBRT for BCS Mastectomy with or without irradiation of the chest wall ART: all three axillary levels and the medial part of the supraclavicular fossa	10-year results Overall survival: 84.6% (ALND) *versus* 81.4% (ART) Disease-free survival: 75.0% (ALND) *versus* 70.1% (ART) Axillary recurrence rate: 0.93% (ALND) *versus* 1.82% (ART)
OTOASOR	cT ≤3 cm cN0 pN1(sn) unrestricted number of metastases (mean number 1.36 and 1.17)	BCS 84% Mastectomy 16%	ALND *versus* RNI	WBRT for BCS RNI: all three axillary levels and the supraclavicular fossa	8-year results Overall survival: 77.9% (ALND) *versus* 84.8% (ART) Disease-free survival: 72.1% (ALND) *versus* 77.4% (ART) Axillary recurrence rate: 2.0% (ALND) *versus* 1.7% (ART)
SINODAR-ONE	cT1–T2 cN0 pN1(sn) 1–2 macrometastases	BCS 75% Mastectomy 25%	SLNB-only *versus* ALND	WBRT for BCS Postmastectomy RT in 17.4%; schedule unknown	5-year results Overall survival: 97.8% (ALND) *versus* 98.7% (SLNB-only) Disease-free survival: 95.7% (ALND) *versus* 94.1% (SLNB-only) Axillary recurrence rate: <1% in each arm
IBCSG 23-01	pT1–T2 cN0 pN1(mi)(sn) unrestricted number of micrometastases (99.9% 1–2 micrometastases)	BCS 91% Mastectomy 9%	SLNB-only *versus* ALND	WBRT in 97% of BCS patients	5-year results Overall survival: 97.6% (ALND) *versus* 97.5% (SLNB-only) Disease-free survival: 84.4% (ALND) *versus* 87.8% (SLNB-only) Axillary recurrence rate: <1% (ALND) *versus* 1% (SLNB-only)
SENOMAC	cT1–T3 cN0 pN1(sn) 1–2 macrometastases	BCS 63% Mastectomy 37%	SLNB-only *versus* ALND	WBRT in 95% of BCS patients Nodal target volumes in 89.9% of SLNB-only group and 88.4% of ALND group (Specific RT target volumes or doses not stipulated)	5-year results Overall survival: 92.0% (ALND) *versus* 92.9% (SLNB-only) Recurrence-free survival: 96.6% (ALND) *versus* 96.6% (SLNB-only)

Systemic adjuvant treatment was given according to national guidelines in all trials. RT, radiotherapy; BCS, breast-conserving surgery; SLNB, sentinel lymph node biopsy; ALND, axillary lymph node dissection; WBRT, whole breast radiotherapy; ART, axillary radiotherapy; RNI, regional nodal irradiation.

To date, there is an ongoing debate over whether AUS should be routinely implemented or not. This debate centres on concerns that AUS may lead to overtreatment through ALND in patients with subclinical nodal metastases who would otherwise be eligible for SLN surgery. This issue is particularly pertinent for patients who lack a clear indication for preoperative systemic therapy (PST), such as those with a luminal breast cancer subtype. Recent developments, however, underline the importance of preoperative AUS. First, the recently reported randomized SOUND trial^16^ enrolled only patients whose clinical node negativity was confirmed by a negative preoperative AUS. After a median follow-up of 5.7 years, the omission of SLN surgery avoided surgical axillary staging and was found to be non-inferior to SLN surgery. The INSEMA trial, which was recently published, poses the same question in a slightly broader patient population and had similar results^17^. Second, pretreatment AUS in patients with breast cancer eligible for PST is crucial for identifying, confirming, and marking axillary metastases thereby facilitating post-PST de-escalation of axillary surgery. Third, the identification of subclinical axillary involvement by AUS may change management recommendations in patients with small triple-negative or HER2-positive breast cancer from primary surgery to PST, with the potential benefit of tailored post-neoadjuvant treatment. Finally, AUS may assist not only in differentiating between node-positive and node-negative breast cancer but also in identifying clinically limited nodal disease, typically involving one to two suspicious axillary metastases^40–42^.

According to current National Comprehensive Cancer Network (NCCN) guidelines, patients with biopsy-confirmed non-palpable limited axillary involvement are eligible for SLN surgery, with or without marking of the metastatic node(s)^43^. This approach restages patients who are already staged, specifically aiming to identify those with no more than one to two SLN metastases, in whom completion ALND could be omitted. This strategy, which may be termed ‘upfront targeted axillary dissection (TAD)’, is suggested for clinically node-negative patients. A similar concept of ‘tailored axillary surgery (TAS)’ is currently evaluated in the TAXIS trial for clinically node-positive patients and adds intraoperative palpation to SLN surgery, with or without marking of the metastatic node(s)^44^.

In a recent publication, 73% of patients with AUS-detected, biopsy-confirmed metastases undergoing SLN surgery successfully avoided ALND as no more than two metastatic SLNs were identified^45^. Considering that the median number of SLNs removed is typically between one and two, this result is not surprising. The total nodal burden detected by ALND in patients with one to two AUS-detected and biopsy-proven axillary metastases is, however, significantly higher than for AUS-negative patients with a positive SLN surgery (32% *versus* 11% for pN2–3)^46^. When ALND is omitted, the total nodal burden remains unknown. This raises questions about whether patients with limited axillary involvement on AUS truly correspond to those with one to two SLN(s) with metastases included in key randomized trials regarding pathological nodal stage and oncological outcomes, highlighting the need for further prospective evaluation. In summary, the significance of AUS needs to be considered from an entirely new angle today, as it has become a tool for the identification of patients who may avoid or undergo de-escalation of axillary surgery and/or benefit from PST.

It appears relevant to reflect upon exactly what information needs to be obtained from axillary staging today. From a historical perspective, the role of axillary surgery has evolved from being purely therapeutic to being more and more a staging procedure—yet the exact situation in which axillary surgery still retains a therapeutic value is unclear and may well be a moving target. If a simple binary staging result, that is negative *versus* positive, was sufficient and an ALND had no therapeutic value, a patient with biopsy-confirmed metastases, whether detected by AUS or clinically, would in theory not require any surgical staging at all. Hence, axillary surgery aims to achieve more detailed information on nodal stage. In clinically node-negative patients, some will argue that it is sufficient to differentiate between patients with one to two SLN metastases and those with three or more SLN metastases, as this reflects eligibility criteria in key randomized trials. It is important to remember, however, that the cut-off between one to two positive SLNs and three or more positive SLNs is arbitrary and not based on clinical relevance or biological mechanisms; only the INSEMA trial allowed patients with one to three positive SLNs to be randomized to either completion ALND or its omission, but unfortunately, this second randomization in the trial was closed prematurely due to low accrual. Others will argue that it is more relevant to differ between pN1 (1–3 nodal metastases) and pN2–3 (4 or more nodal metastases), which may necessitate an ALND. Randomized trials did not show inferior survival after omission of ALND despite 12–13% of included patients with pN2–3 status^8,12,33–35^; however, such information still has implications for intensified systemic treatment. The indication for adjuvant abemaciclib in luminal T1–2 breast cancer of grade 1–2, for example, is based on the identification of pN2–3 status and thus requires an ALND, which would unnecessarily expose a disproportionate number of patients to the risk of long-term arm morbidity^15^. This has limited relevance, however, since the recent US Food and Drug Administration (FDA) approval of ribociclib after the publication of the NATALEE trial results^47,48^. Another concern is that the omission of ALND may fail to identify patients with a clear indication for chemotherapy, potentially causing undertreatment. While adjuvant chemotherapy rates were not different in patients enrolled in the TAXIS trial, assigned to ALND or its omission^49^, this was not true for the SENOMAC and SINODAR-ONE trials. In SENOMAC, chemotherapy was significantly less frequently prescribed in postmenopausal patients with luminal breast cancer without a completion ALND in Denmark (31.4% after SLN surgery only *versus* 41.3% after completion ALND)^50^. In SINODAR-ONE, 52.9% of patients assigned to completion ALND received adjuvant chemotherapy but only 44.6% in the SLN surgery only group^35^. So far, no effects on recurrence or survival rates have been observed, but long-term follow-up will be essential. Finally, radiotherapy target volumes may depend on pN2–3 status even though this should be less problematic after the meta-analysis of the Early Breast Cancer Trialists’ Collaborative Group and the recently published Danish Breast Cancer Group Internal Mammary Node Study^51–53^.

Even though current treatment decisions are increasingly driven by tumour biology and the use of genomic profiles even in the node-positive population, information regarding the extent of nodal involvement may still be relevant in some patients^21^. In order not to expose a substantial proportion of patients to unnecessary long-term arm morbidity and reduced patient-reported quality of life^15,54^, without gaining the benefit of more accurate treatment, non-surgical, reliable staging tools must be identified with urgency. The establishment of predictive nomograms with potential support from deep-learning algorithms may aid in this quest and offer patients accurate risk assessment to base adjuvant treatment decisions upon without risking debilitating physical and functional consequences of extensive axillary surgery^55^. To this end, a predictive nomogram for high nodal burden was recently published, which is currently being externally validated and will soon be available as an online tool for decision support^55^.

### Part 3—De-escalation of axillary surgery after neoadjuvant chemotherapy (NAC)

Anna Weiss and Judy C. Boughey

#### Background

NAC was initially used to downsize breast tumours to make inoperable tumours operable, or to increase success of breast conservation. The National Surgical Adjuvant Breast and Bowel Project (NSABP) B-18^56^ and European Organization for Research and Treatment of Cancer 10902^57^ trials randomized women with breast cancer to NAC followed by surgery, *versus* surgery followed by adjuvant chemotherapy, and demonstrated an increase in breast conservation rates with no difference in survival outcomes.

Additionally NAC can result in eradication of nodal disease, with a 44% nodal pCR rate in NSABP B-18 among initially node-positive patients^58^. In the NSABP B-27 trial in which preoperative or postoperative docetaxel was added to doxorubicin and cyclophosphamide, 179 participating surgeons attempted SLN surgery before ALND in at least one patient. The success rate of SLN surgery was 84.8%, the overall accuracy was 95.6%, and the FNR was 10.7%^59^. These trials predated current understanding of tumour biology, targeted therapies, and patient selection for NAC, so overall and nodal pCR rates were lower than contemporary regimens. Still, these early clinical trials initiated the evolution of axillary surgery for patients treated with NAC.

#### Clinically node-negative, pathologically node-negative patients

Patients who present with a clinically negative axilla (cN0) before NAC comprised the first group of patients where axillary surgery de-escalation after NAC was considered. The initial debate centred on appropriate timing of SLN surgery. Performing SLN surgery before NAC was thought to more accurately stage the axilla due to the absence of potential treatment effect on lymphatic mapping^60^; furthermore, it provided clear information on nodal burden at diagnosis to guide adjuvant radiation. However, upfront SLN surgery leads to a higher rate of pathological node-positive (pN+) disease and thus a higher rate of ALND^61,62^. Most importantly, it does not allow assessment of response to therapy, which over time was shown to be critical prognostic information gained from NAC and is now used to adjust adjuvant therapies. A large retrospective single institution series of 3746 patients who underwent SLN surgery (3171 before NAC and 575 after NAC) between 1994 and 2007 showed that SLN surgery was feasible after NAC and there were fewer patients with pN+ disease with the use of SLN surgery after NAC, thus decreasing ALND^61^. It also demonstrated equivalent locoregional recurrence (LRR) between groups. In the GANEA2 trial, 419 cN0 patients were treated with SLN surgery alone, and there was only one axillary recurrence during the median 36-month follow-up interval^63^. These data led to the increased adoption of SLN surgery after NAC for patients with cN0 disease at presentation, with omission of ALND for patients with negative SLN(s) and reserving completion ALND for patients with any nodal disease at surgery after NAC.

#### Clinically node-positive, pathologically node-negative patients

As systemic therapy advanced (with the addition of taxanes, HER2-targeted therapy, and most recently immunotherapy), rates of nodal pCR after NAC for cN+ disease increased and led to questioning whether SLN surgery could be used to identify patients with eradication of nodal disease (ypN0 disease) and allow omission of ALND in these patients. Several practice-changing clinical trials evaluated SLN surgery followed by ALND among cN1 patients treated with NAC, and reported technical factors that could reduce the FNR of SLN surgery to an acceptable level^59,64,65^, as shown in *Table 2*. A subset analysis of ACOSOG Z1071 revealed that among patients with pre-NAC lymph node clip placement, if the clipped lymph node was resected among the SLN specimens, the FNR was lower at only 6.8%^66^. TAD was developed^67^, which refers to SLN surgery along with ensuring resection of the clipped node with preoperative localization. These trials have led to widespread adoption of SLN surgery after NAC, with or without TAD, mostly among patients who present with cN1 disease and demonstrate response to NAC, the goal being to identify patients with eradication of axillary disease and enable de-escalation of surgery. It is important to note that ALND is recommended for patients with disease progression, lack of response to NAC, or imaging indicating large volume residual nodal disease. The trials focused on cN1 disease and thus data are lacking on SLN surgery for patients who present with cN2 or N3 disease. If cN2 disease is due to positive internal mammary nodes, then axillary management strategies similar to cN1 disease seem appropriate^68^. For patients with cN2 disease with fixed matted nodes at presentation, the standard recommendation is ALND, although SLN surgery can be considered on a case-by-case basis taking into account tumour biology, response in the breast and axilla, imaging findings post-NAC, and multidisciplinary discussion.

**Table 2:** Factors influencing FNRs in landmark clinical trials and smaller prospective series showing that SLN surgery is acceptable among cN1 patients after NAC

Method to reduce FNR	Study	Method details	FNR (%)
Overall trial results	NSABP B-27^59^	No protocol requirements, at least 1 SLN removed	10.7*
	GANEA 1^63^	Dual tracer required, 1 or more SLNs removed	15*
	Z1071^66^	Dual tracer encouraged, 2 or more SLNs required	12.6
	SENTINA^62^	Radioisotope required, 1 or more SLNs removed	14.2
	SN FNAC^71^	Radioisotope required, 1 or more SLNs and IHC	8.4
SLN mapping agent	NSABP B-27^59^	Lymphazurin blue Radiocolloid Dual agent	14* 5* 9.3*
	Z1071^66^	Single agent Dual agent	20.3 10.8
	SENTINA^62^	Single agent Dual agent	16 8.6
	SN FNAC^71^	Single agent Dual agent	16 5.2
Number of SLNs obtained	NSABP B-27^59^	1–4 nodes 5 or more nodes	12.3* 9.0*
	Z1071^66^	2 nodes 3 or more nodes	21.1 9.1
	SENTINA^62^	1 node 2 nodes 2 or more nodes 3 or more nodes	24.3 18.5 9.6 7.3
	SN FNAC^71^	1 node 2 or more nodes	18.2 4.9
Pathological evaluation	Z1071^66^	H&E IHC	11.3 8.7
	SN FNAC^71^	H&E IHC	13.3 8.4
Localization/identification of the clipped node	Z1071^66^	Retrieval of clipped node as SLN verified in pathological specimen	6.8
	MARI^173^	Radioactive seed placed at time of pretreatment LN biopsy removed	7
	TAD^67^	Clipped node retrieved with radioactive seed localization plus SLN surgery	2.4
	Diego *et al*.^174^	TAD	0
	ILINA^175^	Intraoperative ultrasound-guided clipped LN retrieval	4.1
	Hartmann *et al*.^19^	Wire localization plus SLN surgery	0
	Cabioglu *et al*.^65^	Dual tracer and retrieval of clipped node as SLN verified in pathological specimen	4.2
	Flores-Funes *et al*.^176^	TAD	0
	Khallaf *et al*.^177^	Carbon tattooing at time of pretreatment LN biopsy, plus SLN surgery with blue dye	8.3
	RISAS^173^	MARI plus SLN surgery	3.5
	SenTA^178^	Various methods including wire, intraoperative ultrasound guidance, or palpation	7.2

^*^Included cN0 and cN1. FNR, false negative rate; SLN, sentinel lymph node; NAC, neoadjuvant chemotherapy; IHC, immunohistochemistry; H&E, hematoxylin and eosin; LN, lymph node; TAD, targeted axillary dissection.

#### Clinically node-negative or node-positive, pathologically node-negative patients

Among patients presenting with either cN0 or cN1 disease, if SLN surgery is performed and the SLNs are negative (ypN0 disease) after NAC, ALND should be omitted. Although there has not been a prospective randomized trial in this space, evidence is mounting that this practice is safe. In a single institution study of 602 total patients, 159 pre-NAC cN1 patients were treated with SLN surgery alone and found to be ypN0, and the 2-year freedom-from-regional-recurrence rate was 99.1%^68^. The largest series thus far is an international retrospective series of 1144 patients with cN1 disease who converted to ypN0 after NAC and underwent SLN surgery only (with or without clipped node localization). Five-year axillary recurrence, LRR, and any invasive recurrence were 1.0%, 2.7%, and 10% respectively^69^. SLN surgery (±TAD) without ALND should now be standard for cN0 and cN1 patients with a good response to NAC who are ypN0, and examining practice patterns reveals this is the case. Among 1578 patients treated on the I-SPY2 trial between 2011 and 2021, 61.7% of patients were treated with SLN surgery alone, with the shift from ALND to SLN surgery alone particularly striking among cN1 patients^70^.

#### Clinically node-negative or node-positive, pathologically node-positive patients

The story is different for patients with positive SLN(s) after NAC (ypN+ disease)—the current standard is ALND. However, this standard is being challenged. One of the major factors limiting omission of ALND for patients with ypN+ disease is the presence of higher volumes of nodal disease, and that this is nodal disease has shown resistant to systemic therapy. Rates of additional positive nodes on ALND in patients with positive SLN(s) after NAC are around 38% in patients with SLN ITCs and 50–60% in patients with SLN macrometastases^71,72^. For cN0 patients with ITCs or micrometastases^73^, experts at the St Gallen consensus conference in 2021 suggested that ALND should be omitted^74^. Retrospective data are emerging that support this opinion and, while there may be more residual nodal disease, the question is whether this impacts outcomes. In an international retrospective study of 583 cN0–3 patients with residual ITCs after NAC, 30% of the 182 patients who underwent ALND had additional positive non-SLNs but there were no differences in oncological outcomes between patients who underwent ALND and those that did not^69^. A multicentre study from Korea (KROG 21-06) compared 170 ypN+ patients who underwent SLN surgery alone with 1103 ypN+ patients who underwent ALND and at a median follow-up of 75.3 months reported similar axillary recurrence rates of 4.7% and 4.8% respectively^75^. Retrospective evaluation of patients treated in the I-SPY2 clinical trial showed a shift away from the use of ALND in patients with ypN+ disease with rates of ALND decreasing from 69% in 2011 to 39% in 2021^70^. Axillary recurrence rates at 5 years were 5.2% in patients without ALND and 3.6% in patients with ALND (*P* = 0.8)^76^. Omission of ALND was not associated with inferior LRR, distant recurrence-free survival, or event-free survival. However, the recently reported OPBC-07/microNAC study^77^ evaluated 1585 patients with micrometastasis (ypN1mi) after NAC, out of which 50.7% underwent completion ALND and 49.3% did not. The rate of additional nodal metastases in those undergoing ALND was 30.5%. While in the overall cohort there was no difference in axillary recurrence between those with ALND and those selected for omission of ALND, among those with TNBC the axillary recurrence rate at 5 years was significantly higher in those with omission of ALND (at 9%) than in those with ALND (at 4%) (*P* = 0.018). Thus, while studies so far make the practice of omitting ALND among ypN+ patients appear safe, there is significant selection bias in which patients are selected for omission of ALND that cannot be controlled for in these retrospective analyses. For example, the nodal burden on pre-NAC and/or post-NAC/preoperative imaging is not known and heavily influences surgeons’ decision-making and multidisciplinary discussions. As these are not level I randomized trial data, until the results of Alliance A011202 (NCT01901094)^78^ and TAXIS (NCT03513614) are available, it is not safe to routinely omit ALND for ypN+ patients.

#### Looking ahead

Patients undergoing primary surgery who are eligible for omission of axillary surgery are increasing; however, this is not yet standard after NAC. An exciting possibility for patients with cN0 disease treated with NAC is the potential to omit axillary surgery altogether, based on data showing very low rates of pathological nodal involvement (<2%) in patients with cT1–2 cN0 HER2+ breast cancer or TNBC who achieve a breast pCR^79,80^, as shown in *Table 3*. The MDACC exceptional responders trial^81^ enrolled 50 patients with unifocal TNBC or HER2+ breast cancer who underwent NAC followed by imaging and percutaneous tumour bed biopsy; 31 patients had no evidence of residual disease on biopsy and had omission of breast surgery and 5-year follow-up showed no in-breast recurrences. EUBREAST-01 (NCT04101851) and ASICS (NCT04225858) trials are also underway: EUBREAST-01 is treating cN0 patients with NAC followed by lumpectomy alone and awaiting final pathology to determine the need for SLN surgery, omitting SLN surgery if there is a breast pCR; and ASICS is omitting SLN surgery among cN0 patients with a complete breast response by MRI. The high response rates of TNBC and HER2+ disease to NAC make omission of SLN surgery a real possibility in the future; however, because these high-risk subtypes have such a clear indication for escalation of adjuvant systemic therapy in the setting of residual disease^82,83^ with associated survival benefit, the oncological safety and impact on systemic therapy will be critical to evaluate.

**Table 3:** Rates of pathological nodal (ypN) disease after NAC among cN0 patients, based on in-breast response

Study	Year	Setting	Number (cN0)	Breast cancer subtypes included	cT category included	Overall ypN0 rates	ypN0 rates based on breast pathology
Tadros *et al*.^79^	2017	Single institution Retrospective	290	Any HR/HER2+ and TNBC	T1–2	96.6% (280/290)	Breast pCR—100% (116/116) Breast RD—94.3% (164/174)
Barron *et al*.^179^	2018	National data set Retrospective	18 093	HR+/HER2−, HR+/HER2+, HR−/HER2+, TNBC	T1–2	85.1% (15 394/18 093)	Breast pCR—98.2% (5912/6023) Breast RD—78.6% (9482/12070)
Choi *et al*.^180^	2019	Single institution Retrospective	200	HR+/HER2−, HR+/HER2+, HR−/HER2+, TNBC	T1–3	81% (162/200)	Breast pCR—96.4% (54/56) Breast RD—75% (108/144)
Samiei *et al*.^181^	2020	Multi-institution Retrospective	1674	HR+/HER2−, HR+/HER2+, HR−/HER2+, TNBC	T1–3	78.5% (1314/1674)	Breast pCR—97.7% (432/442) Breast RD—71.6% (882/1232)
Zhu *et al*.^182^	2020	Multi-institution Retrospective	406	HR+/HER2−, HR+/HER2+, HR−/HER2+, TNBC	T1–4	63.5% (258/406)	Breast pCR—89% (76/85) Breast RD—57% (182/321)
Weiss *et al*.^183^	2021	Multi-institution RCT Unplanned analysis	241	Any HR/HER2+ and TNBC	T1–4	88.8% (214/241)	Breast pCR—96.3% (104/108) Breast RD—82.7% (110/133)
Shi *et al*.^184^	2021	Single institution Retrospective	37	Any HR/HER2+ and TNBC	T1–4	94.6% (35/37)	Breast pCR—100% (17/17) Breast RD—90% (18/20)
Hong *et al*.^185^	2021	Multi-institution Retrospective	1999	Luminal A, luminal B (HER2− and HER2+), HER2+, TNBC	T1–4	45.9% (917/1999)	Breast pCR—76.1% (331/435) Breast RD—37.5% (586/1564)
Esgueva *et al*.^186^	2021	Two institutions Retrospective	265	Luminal A, luminal B HER2−, HER2+, TNBC	T1–4	80.7% (214/265)	Breast pCR—94.1% (64/68) Breast RD—76.1% (150/197)
Ryu *et al*.^187^	2022	Multi-institution Retrospective	1529	HR+/HER2−, HR+/HER2+, HR−/HER2+, TNBC	T1–3	83.1% (1271/1529)	Breast pCR—99% (301/304) Breast RD—79.2% (970/1225)
Weiss *et al*.^80^	2024	Multi-institution clinical trial Exploratory analysis	92	Any HR/HER2+	T1–3	91.3% (84/92)	Breast pCR—100% (48/48) Breast RD—81.8% (36/44)

NAC, neoadjuvant chemotherapy; cT, clinical tumour; ypN0, node negative at pathology after neoadjuvant therapy; HR, hormone receptor; HER2, human epidermal growth factor receptor 2; TNBC, triple-negative breast cancer; RD, residual disease.

#### Summary

It is anticipated that as NAC regimens (along with targeted therapies and immunotherapy for appropriate tumour subtypes) continue to evolve, and understanding of which tumours benefit most from NAC grows, nodal pCR rates will continue to rise, and axillary surgery after NAC will continue to evolve as well. Currently, SLN surgery should be attempted after NAC for all patients with cN0 disease and most with cN1, especially those who exhibit a response to NAC. If the nodes are negative, ALND should be omitted. If the nodes are positive and after multidisciplinary considerations including nodal metastasis volume, tumour biology, radiation plans, and patient preference, most should undergo ALND until clinical trial results prove otherwise.

### Part 4—Strategies to minimize the morbidity of breast cancer-related lymphoedema (BCRL)

Paul T. R. Thiruchelvam, Anna Duncan, Meera Joshi and Maria Mani

#### Lymphoedema pathophysiology and clinical presentation

BCRL is a common, chronic, debilitating, and incurable condition that is often under-reported and undertreated^84^. It is characterized by persistent and progressive symptoms including discomfort, heaviness, pain, recurrent infections, and functional limitation. It results from disruption of the lymphatic system and represents a major burden to both the patient and the healthcare system including a negative quality of life due to loss of function^85,86^. Lymphoedema develops because of lymphatic dysfunction and is characterized by ectasia of lymphatic vessels leading to valve dysfunction with resultant reflux of lymphatic fluid into the interstitial space^87^. Lymphatic vessels initially become dilated, lymphatic fluid leaks into the interstitium, and a T cell-mediated immune response is triggered, causing fibrosis of the lymphatic vessels, impaired function, and adipose deposition in the subcutaneous space in the chronic stage^88^. Whilst primary lymphoedema is caused by a developmental abnormality in the lymphatic anatomy, secondary lymphoedema, including BCRL, results from underlying disease, infection, trauma, or iatrogenic injury.

#### Early screening and diagnosis

Early detection of BCRL is challenging due to heterogeneity of screening protocols, subtlety in the initial onset of symptoms, and subjectivity of clinical assessment measures^89^. Multiple assessment modalities that aid in screening, diagnosis, and treatment planning are outlined in *Table 4*^90^. Bioimpedance analysis (BIA) and bioimpedance spectroscopy (BIS) measure the electrical impedance of tissue, which serves as a measure for fluid and fat composition, which is key for early detection of subclinical lymphoedema in high-risk patients^91^. Lymphoscintigraphy is the ‘gold standard’ for diagnosing lymphoedema as it provides an anatomic assessment of the lymphatic flow patterns and the presence and function of locoregional nodes^92^. Indocyanine green (ICG) lymphography is an invaluable tool for preoperative planning, diagnosis, and staging of established lymphoedema and for intraoperative visualization during lymphovenous anastomosis (LVA) and immediate lymphatic reconstruction (ILR). Ultra-high frequency ultrasound (UHFUS) has recently gained popularity as it enables visualization of submillimetre lymphatic vessels and nearby vein targets for LVA planning; however, depth is limited, making assessment of deeper vessels challenging^93^. Surveillance programmes are recommended for those at high risk of BCRL.

**Table 4:** Clinical objective measures of lymphoedema

Modality	Mechanism	Advantages	Disadvantages
Circumferential arm measurements	Simulated tape measurements every 4–5 cm from the wrist to axilla Volumes calculated by the sum of truncated cones (frustrum) model	Reliable with extensive training Inexpensive Easily accessible	Time-consuming and cumbersome Requires rigorous training to achieve reproducible results Inter/intra-rater variability
Water displacement	Arm is placed in a cylinder of water, any displaced water is measured and compared with contralateral side	Accurate Inexpensive Reliable and validated Includes the hand	No swelling localization information Contraindicated if open wounds Hygienic concerns
Perometry	Frame of infrared light beam–receiver pairs to measure limb outline with subcentimetre definition and thus derive limb volume by the disc model method	Rapid measurement Highly reproducible Can measure bilateral BCRL Can detect 3% limb volume change Ability to measure torso with some models	Expensive Equipment not available everywhere Difficulty with reliable hand measurements Positioning can be problematic
Bioimpedance spectroscopy	Impedance ratio between limbs Calculates L-Dex ratio	Rapid, painless testing Repeatable results Early detection possible (subclinical lymphoedema) High specificity	Expensive Limited bilateral limb involvement interpretations Limited to arm and leg use
3D laser scanning	Real-time digital 3D image	Sensitive to small variations in arm volume	Expensive Arm reference point difficulties Reliability uncertain
Lymphoscintigraphy	Axillary/elbow lymph nodes Lymphatic ducts Dermal backflow	Objective volume measurement	Protocol not standardized Potential for poor image quality Little subdermal lymphatic information
ICG-lymphography	Superficial lymphatic ducts Dermal backflow	Detailed visualization Intraoperative measurement No radiation exposure	Limited to lymphatics ∼2.0 cm deep in subcutaneous tissue Not available everywhere Technically demanding Time-consuming
CT	Skin thickening, honeycombing Fat lobules	Objective volume measurement	Expensive Exposure to radiation
MRI lymphangiography	Lymphatics Fat deposition Muscle compartments Precise limb volume	Provides information on lymphatic function No radiation exposure	Expensive Not available everywhere Technically demanding
Ultra-high frequency ultrasound	Lymphoedema evidenced by fluid in subcutaneous space Lymphatics identified as irregular hypoechoic lumens, compressible, with no colour on Doppler mode and no convergence to nearby vessels	No radiation Outpatient setting Identifies depth of lymph vessels and nearby veins for LVA	Operator dependent Difficult to distinguish from vessels/nerves Depth limited

BCRL, breast cancer-related lymphoedema; 3D, three-dimensional; ICG, indocyanine green; LVA, lymphovenous anastomosis.

#### Identification of risk factors

The incidence of BCRL varies widely but has been reported to be as high as 56%^94^. There are well-established patient-specific and treatment-specific risk factors (*Table 5*). Independent treatment-related risk factors include use of taxane chemotherapy, axillary lymph node surgery, and regional nodal irradiation (RNI) (*Table 5*). Preoperative screening for lymphoedema risk factors and subclinical lymphoedema is crucial to identify patients at high risk of BCRL and mitigate the morbidity associated with their cancer treatment. It is acknowledged that the patients still requiring ALND are those with the highest burden of disease where surgical de-escalation is not appropriate and have the greatest risk. Recently, prophylactic use of compression sleeves after surgery has been demonstrated to reduce arm swelling in women at high risk of lymphoedema^95^.

**Table 5:** Patient- and treatment-specific risk factors for BCRL

Patient-specific factors	Treatment-specific factors
BMI	Axillary surgery
Cellulitis	Regional lymph node radiation
Subclinical oedema	Mastectomy
Racial origin	Breast reconstruction
Age	
Genetics	

BCRL, breast cancer-related lymphoedema.

#### Surgical prevention strategies

Several approaches for surgical prevention of BCRL as a sequela of axillary surgery have been developed in recent years.

(1) Axillary de-escalation

Recent trials support axillary de-escalation in breast cancer, such as ACOSOG Z0011^32^, AMAROS^11^, OTOASOR^34^, and SOUND^16^. These trials demonstrate that patients with low-burden nodal disease or early-stage disease can either safely avoid ALND or avoid axillary surgery altogether. TAXIS is an ongoing international study investigating de-escalation of axillary surgery in node-positive patients in the upfront surgical setting^44^.

(2) Radiotherapy de-escalation

RNI significantly increases the risk of BCRL when administered with ALND (33.4%), compared with ALND alone (14.1%)^96^, and several trials are assessing radiotherapy de-escalation. Canadian Cancer Trials Group MA.39 TAILOR RT is a randomized phase III study assessing omission of locoregional radiation in ER+/HER2− patients with low risk for recurence based on biomarker analysis (recurrence score <18) and low-burden nodal disease. The Alliance A11202 trial is evaluating adjuvant axillary radiotherapy compared with ALND in patients with residual nodal disease after NAC. The NRG/NSABP B-51/RTOG 1304 trial (NCT01872975) demonstrated the safe omission of RNI in patients with T1–3 N1 disease who became node negative after NAC.

(3) Axillary reverse mapping (ARM)

ARM involves injection of a blue or fluorescent dye into the ipsilateral arm at time of axillary dissection. Lymphatic mapping enables an oncological surgeon to preserve the visible lymphatics and/or nodes; however, if a lymphatic is transected, it may be used for lymphatic approximation^14^. In a large, prospective study of ARM, the technique reduced lymphoedema incidence for SLN surgery and ALND to 0.8% and 6.5% respectively at 2-year follow-up^97^.

(4) ILR/lymphatic microsurgical preventive healing approach (LyMPHA)

**Fig. 1:**
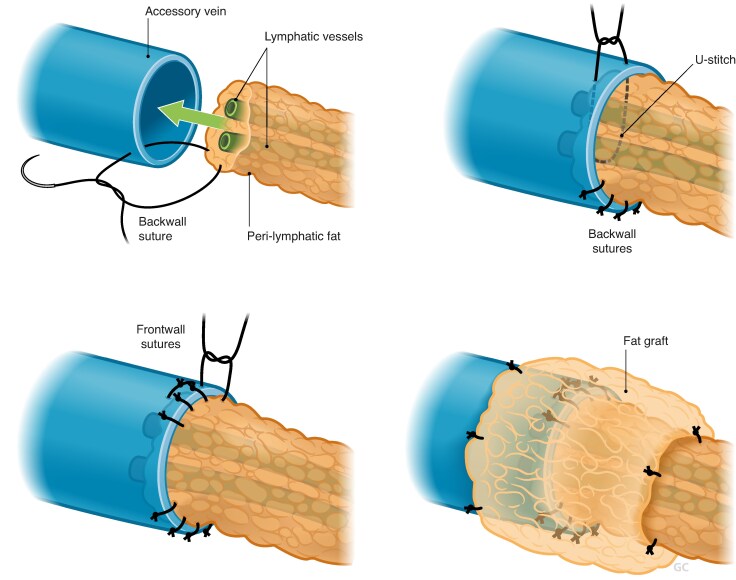
Lymphovenous bypass technique used in immediate lymphatic reconstruction (ILR) showing multiple small lymphatics being “dunked in” to a larger vein with a U-stitch and secured with perilymphatic sutures to the vessel lumen Optional fat graft covering anastomosis.

**Fig. 2:**
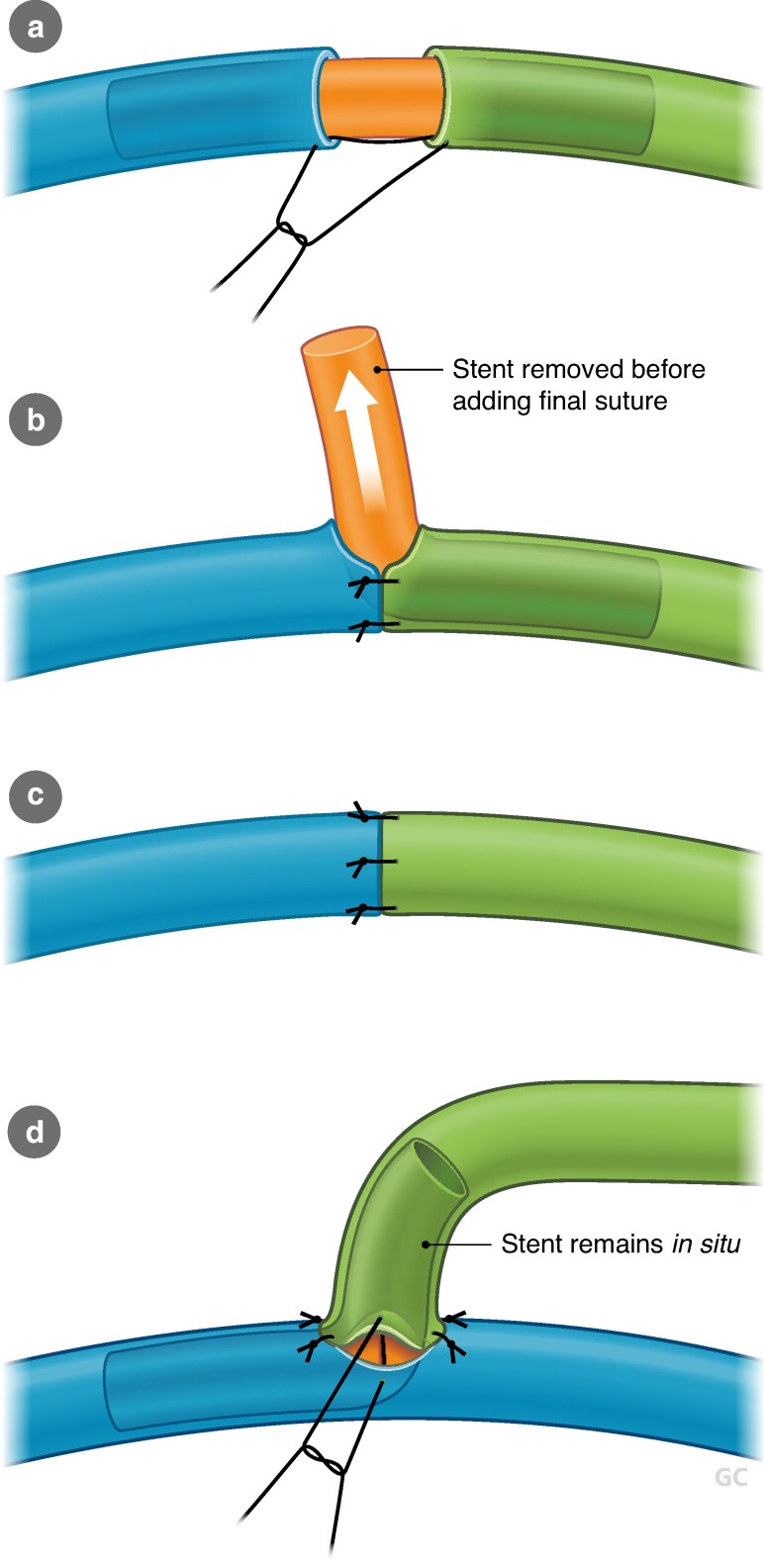
Lymphovenous anastomosis (LVA) technique using supermicrosurgery lumen-to-lumen technique and high-powered magnification **a-c** Vein (blue) sutured end-to-end with lymphatic (green). **d** Lymphatic (green) sutured end to side with vein (blue). The anastomosis is typically performed using a 10-0 or 11-0 nylon microsuture.

ILR, also referred to as LyMPHA, represents an evolution of ARM in which the transected lymphatic channels draining the ipsilateral arm are immediately ‘bypassed’ to local recipient veins in the axilla. There is variation in the anastomotic techniques employed, including multiple lymphatics ‘dunked into’ a single vein (microsurgery)^98^, a lumen-to-lumen technique using an end-to-end or end-to-side anastomosis (supermicrosurgery)^99^, and use of coupler-assisted devices, allowing for smaller lymphatics to be anastomosed to larger-calibre veins^100^ (*Fig. 1* and *Fig. 2*). No studies have yet demonstrated the superiority of one technique over another. LVA or lymphovenous bypass at the time of ALND is not associated with higher risks of reoperation or wound complications; however, it does increase operating time. Benefits of ILR include recruitment of larger-calibre lymphatic vessels, avoidance of secondary surgery, and prevention of the sequelae of BCRL. This is compared with LVA performed for established lymphoedema, which is performed distally and with smaller vessels with some degree of fibrosis, making dissection and the anastomosis more challenging. Although head-to-head comparisons of ILR and non-ILR groups are few, several studies have suggested that ILR is feasible, safe, and effective in the primary prevention of BCRL. A summary of these studies is shown in *Table 6*. In a recent meta-analysis of 11 studies and 417 patients, BCRL was reported in 6.7% of the ILR group compared with 34% of the control group^123^. Early results from a recent RCT demonstrated a significant reduction in the incidence of BCRL from 32% to 9.5% in the control group *versus* the treatment group, and several other trials are accruing^124^. A recent, prospective study reported no significant difference in BCRL rates between ILR and non-ILR groups; however, this may be explained by the non-randomized trial design, selection bias, and inconsistent measurement methodology, including EMR documentation of lymphoedema and ICD-10 coding. Furthermore, the majority of the ILR procedures were performed using a non-microsurgical technique (simplified LyMPHA (s-LyMPHA))^120^.

**Table 6:** Studies of ILR/LyMPHA

Study	Country	LyMPHA/s-LyMHPA	Number of patients	Lymphoedema (%)	Follow-up	Method detection	Technical failure rate (%)
Boccardo *et al*.^101^ 2014	Italy	LyMPHA	71	4.1	48 months	Volumetry	NS
Feldman *et al*.^102^ 2015	USA	LyMPHA	46	7.0	30 months	Bioimpedence, clinical assessment, circumferential arm measurement	21.7 (first 25 cases)
Agrawal *et al*.^103^ 2018	India, Dehli	LyMPHA	35	NS	NS	Circumferential arm measurement	0
Johnson *et al*.^104^ 2019	USA, Harvard	LyMPHA	106	2.1 without LRRT 10.3 with LRRT	24 months	Circumferential arm measurement (RVS 10%), bioimpedence	NS
Ozmen *et al*.^105^ 2019	USA, Florida	s-LyMPHA	72	3	15 months (median)	Circumferential arm assessment	NS
Schwarz *et al*.^106^ 2019	USA, Ohio	LyMPHA	58	4.6	12 months	Bioimpedence, clinical assessment, circumferential arm measurement	5
Cook *et al*.^107^ 2020	USA, Indianapolis	LyMPHA	33	9.1	18 months	Limb circumference increase >2 cm	NS
Herremans *et al*.^108^ 2021	USA	LyMPHA	84	13.2	60 months	Bioimpedence, clinical assessment, circumferential arm measurement	9.5—NB Microsurgery done by GS trained by MS
Hahamoff *et al*.^109^ 2021	USA, Florida	LyMPHA	87	5.3	10 months	Bioimpedence, clinical assessment, circumferential arm measurement	0
Lipman *et al*.^99^ 2021	USA, California	LyMPHA	19	12.5	15 months	Bioimpedence, clinical assessment, circumferential arm measurement	0
Weinstein *et al*.^110^ 2022	USA, Florida	LyMPHA	66	6.0	8 months	Circumferential arm measurement, bioimpedance	NS
Levy *et al*.^111^ 2023	USA, New York	LyMPHA	45	31.1	48 months	Bioimpedence, arm circumference	NS
Le *et al*.^112^ 2023	USA, Florida	LyMPHA	252	4.7	NS	Circumferential arm measurements (RVS >5%), bioimpedence	NS
Le *et al*.^113^ 2023	USA, Florida	LyMPHA	77	10	14 months	Bioimpedence, arm circumference, patient-reported symptoms	NS
Coriddi *et al*.^114^ 2023	USA New York	LyMPHA	72	9.5	24 month	Bioimpedence, arm circumference, patient reported, Lymphoedema Quality of Life (LYMQOL), Upper Limb Lymphoedema - 27 (ULL-27)	NS
Mughal *et al*.^115^ 2023	UK, London (Guy’s)	LyMPHA	72	6.25	12.1 months (mean)	Circumferential arm measurement	NS
Chung *et al*.^116^ 2023	Korea, Seoul	LyMPHA	26	3.8	14 months (mean)	Arm circumference	13.3
Granoff *et al*.^117^ 2023	USA, Harvard (Dhruv Singhal team)	LyMPHA	99	9	17 months (median)	Bioimpedence, arm circumference, patient-reported symptoms	NS
Brahma *et al*.^118^ 2024	Indonesia	LyMPHA	82 (only 50 patients followed up)	22	12.5 months	ICG-lymphography	NS
Haravu *et al*.^119^ 2024	USA, Chicago	LyMPHA	90	10.9	24 months	Preoperative and postoperative circumferential limb measurements, volumetric limb measurements, bioimpedance, validated Disability of Arm, Shoulder and Hand questionnaire (DASH Score)	NS
Jakub *et al*.^120^ 2024	USA, Florida	LyMPHA and s-LyMPHA (1 breast surgeon, others all plastics)	131	>10 no difference *versus* ALND alone	24 months	Circumferential arm measurement, patient self-reporting, provider documentation	NS
Wainwright *et al*.^121^ 2023	USA, Florida	LyMPHA	349	9.4	3 months	Circumferential arm measurement, subjective reporting, clinical assessment	NS
Médor *et al*.^122^ 2024	Canada, Montreal	LyMPHA	42	7.7	18 months	Bilateral upper limb volumes measured using Bronson’s truncated cone formula and the Pero-System (3D Körper scanner), patient-reported quality-of-life questionnaire	NS
Spoer *et al*.^100^ 2024	USA, Washington	LyMPHA	63	Coupler-assisted bypass—9.1 Standard end-to-end suture—4.8	14.7 months	Bioimpedence, arm circumference, patient-reported symptoms	NS

ILR, immediate lymphatic reconstruction; LyMPHA, lymphatic microsurgical preventive healing approach; s-LyMPHA, simplified LyMPHA; NS, not specified; RVS, relative value scale; NB, nota bene; GS, general surgeon; MS, microsurgeon; ICG, indocyanine green; 3D, three-dimensional.

Appropriate patient selection for prophylactic lymphatic reconstruction, optimization of intraoperative imaging, and technical refinements for ILR remain areas of ongoing development, as they vary widely across centres and lack clear consensus. There is a risk of overtreatment with ILR, a resource intensive intervention, given that many of the patients undergoing axillary surgery for breast cancer will not develop BCRL. Further studies are needed to identify the patients at greatest risk of BCRL and as it is these that will benefit the most from preventative surgery, thereby optimizing incidence reduction and enabling better personalized care.

**Fig. 3:**
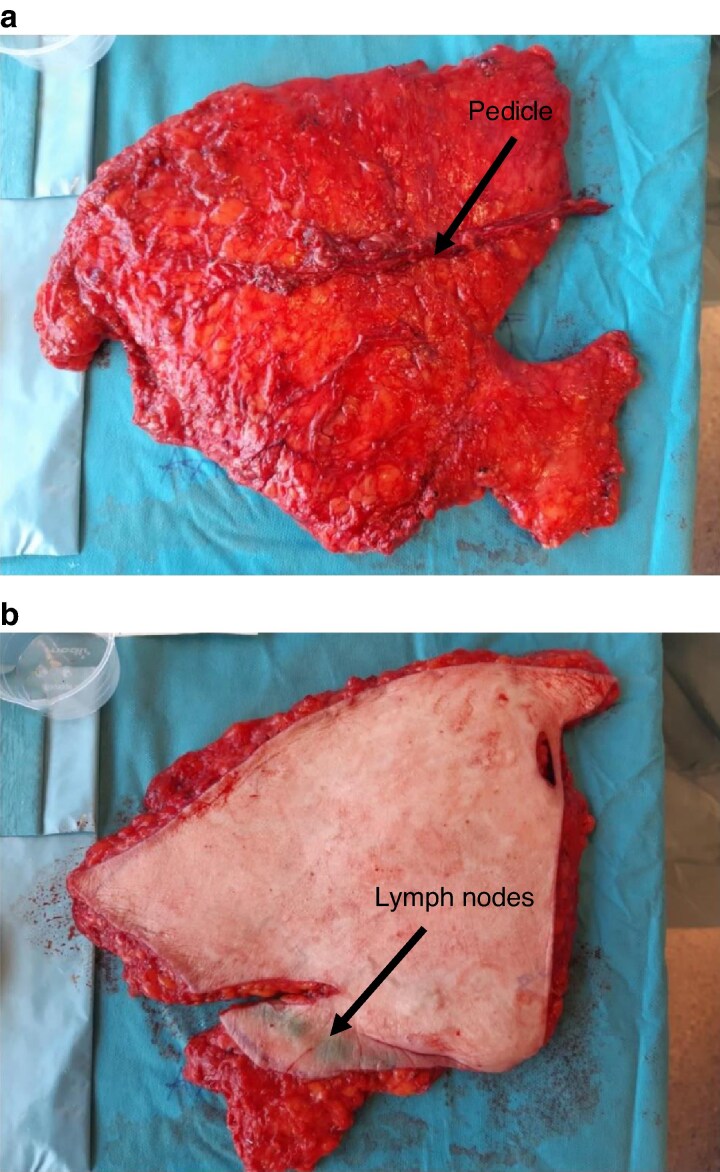
Abdominally based DIEP fasciocutaneous flap harvested with vascularized inguinal lymph nodes **a** Posterior view showing the perforator and dissected vascular pedicle where it enters the underside of the flap. **b** Anterior view showing where the ICG has been injected into the dermis to identify the inguinal lymph nodes to be included in the flap harvest. DIEP, deep inferior epigastric artery perforator; ICG, indocyanine green.

Other techniques for lymphatic reconstruction in the setting of breast cancer and axillary dissection include the total breast autologous reconstruction (TBAR) technique, which includes simultaneous autologous breast reconstruction and vascularized lymph node transfer (vLNT) (*Fig. 3*)^125^. This technique has been primarily described and investigated in the setting of delayed reconstruction in those patients with established lymphoedema, but immediate reconstruction with concurrent vLNT is an area of future research.

#### Non-surgical treatment for lymphoedema

**Fig. 4:**
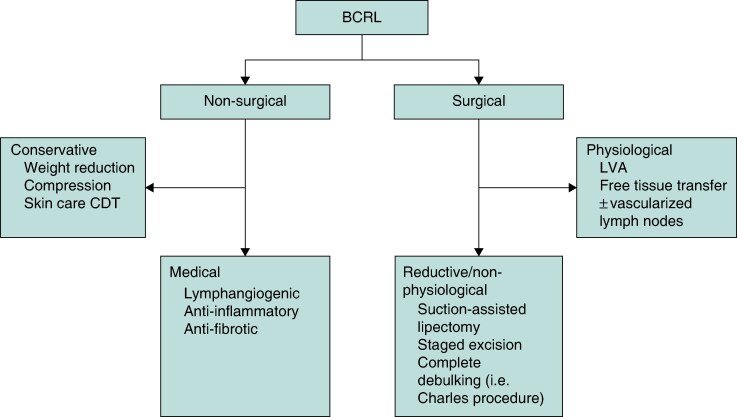
Major approaches to management of established BCRL including non-surgical and surgical methods BCRL, breast cancer-related lymphoedema; CDT, complete decongestive therapy; LVA, lymphovenous anastomosis.

For patients with established lymphoedema, there are both non-operative and operative management options (*Fig. 4*). Combined decongestive therapy (CDT) is the non-operative standard of care for acquired lymphoedema and consists of manual lymphatic drainage (MLD), gradient compression bandaging (GCB), therapeutic exercise, and skin care. Patient education is a critical aspect of BCRL prevention. Patient-reported symptoms (that is arm heaviness and ill-fitting jewellery/clothing) often herald early lymphoedema and can be the trigger for early intervention including compression, which is superior in efficacy to late intervention^126^. Cellulitis may act as a trigger for BCRL and, as such, patients should be cautioned to take infection prevention measures.

#### Surgical treatment for established lymphoedema

For BCRL that does not respond to conservative management, several surgical options are well established. These are traditionally divided into physiological (reconstructive) and non-physiological (reductive) options, where physiological surgery is typically reserved for those with reversible lymphoedema and non-physiological techniques, such as liposuction and debulking, are used in cases where the lymphatic fluid has converted into fixed fibroadipose tissue (*Fig. 4*). The efficacy of suction-assisted lipectomy combined with compression for sustained volume reduction, increased quality of life, and lower incidence of cellulitis has been demonstrated in long-term cohort studies^127^.

**Fig. 5:**
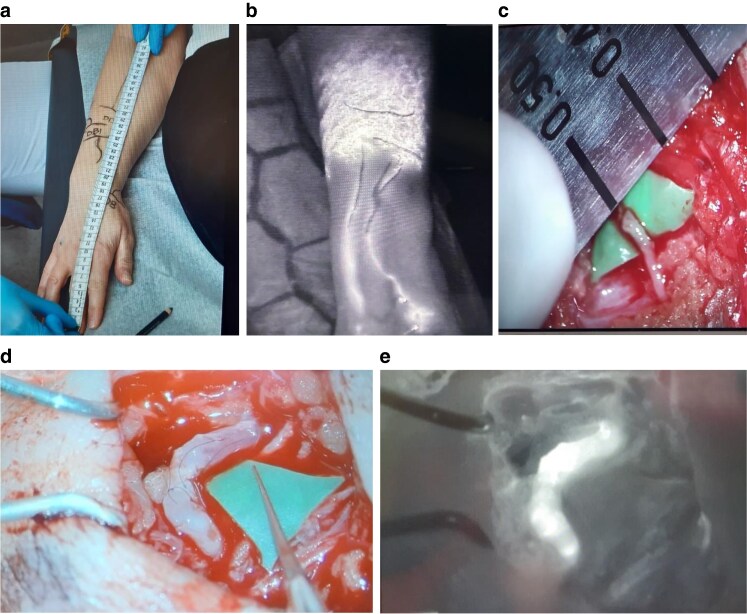
Preoperative and intraoperative techniques for distal extremity LVA **a** and **b** ICG is injected distally in the web spaces and the lymphatic flow is visualized with an infrared camera. The distance is measured and the transition points marked between linear flow, which denotes functional lymphatics, and dermal backflow. This will be the location for the LVA. **c** Intraoperatively, the typically submillimetre lymphatics are dissected free and transected proximally. **d** They are then anastomosed either end to end or end to side with a nearby vein. **e** Flow across the anastomosis is confirmed with ICG and infrared imaging once again. LVA, lymphovenous anastomosis; ICG, indocyanine green.

The aim of an LVA is to divert lymphatic fluid from the lymphatic system into the venous system thereby lowering the fluid burden on the damaged lymphatic system. Before surgery, patients are assessed with ICG-lymphography (ICG-L) to visualize and identify the transition point between the patent lymphatics and damaged vessels. This often correlates to the transition from a linear vessel pattern to dermal backflow on the ICG-L. At this site the patent lymphatic vessel is anastomosed to a nearby recipient vein (*Fig. 5*). The optimal timing of surgical intervention, sites for anastomosis, specific techniques used, number of anastomoses, postoperative regimens, and conjunct procedures remain active areas of investigation and debate.

A further physiological treatment option is vLNT, which has demonstrated efficacy for established lymphoedema^128^. It involves the transfer of functional lymph nodes, with microanastomosis of vasculature in the recipient bed, to restore lymphatic flow to a region in which the lymph nodes have been excised. Retrospective studies have shown similar outcomes for both LVA and vLNT, with a higher risk of complications for vLNT including risk of donor site morbidity^129^. Prospective comparison of these two microsurgical treatments is ongoing. Furthermore, vascularized lymph nodes from the groin can also be transferred concomitantly with free abdominally based autologous tissue during delayed breast reconstruction, as described previously (*Fig. 3*).

#### Future therapies for BCRL

**Fig. 6:**
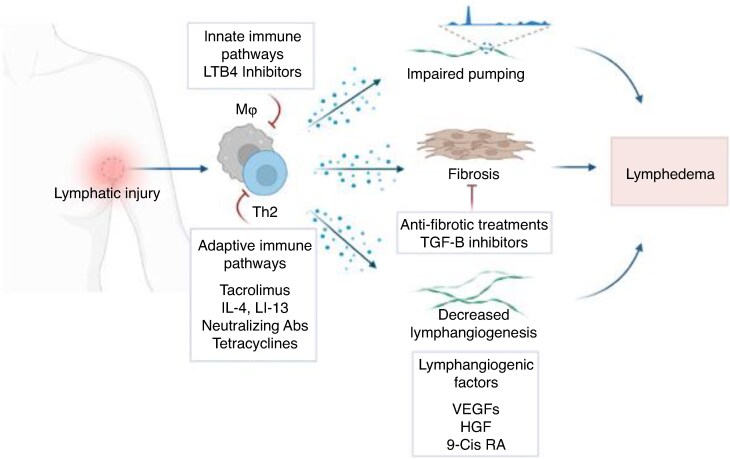
Pharmacological treatment of secondary lymphoedema Reproduced under a Creative Commons CC BY 4.0 Deed from Brown S, Dayan JH, Coriddi M, Campbell A, Kuonqui K, Shin J *et al*. Pharmacological treatment of secondary lymphedema. *Front Pharmacol* 2022;**13**:828513.

Developing effective treatment strategies for lymphoedema remains an important unmet clinical need. The main therapeutic strategies for the treatment of lymphoedema consisting of lymphangiogenic interventions, anti-inflammatory treatments, and anti-fibrotic agents are summarized in *Fig. 6*. These treatments may be used in combination with traditional methods of treating lymphoedema or as an adjunct to surgical management. A recent retrospective review of BCRL in patients taking glucagon-like peptide-1 receptor agonists (GLP-1 RAs) highlighted an 86% reduction in BCRL in those patients treated with a GLP-1 RA^130^. Proposed mechanisms of action include: central suppression of appetite and delayed gastric emptying resulting in weight loss; a protective effect on the lymphatic system via improved insulin signalling essential for lymphangiogenesis and endothelial cell metabolism; and inhibition of both cellular and humoral key mediators in the development of lymphoedema including CD4+ cell migration and interleukin 5, interleukin 13, and transforming growth factor β1 signalling. A summary of pharmacological interventions for the treatment of lymphoedema is presented in *Table 7*.^131^

**Table 7:** Novel pharmacological interventions for the treatment of lymphoedema

Proposed mechanism for lymphedema treatment	Intervention type
Lymphangiogenesis	(i) VEGF-C *Subcutaneous Injection* – binds VEGFR-3 that promotes LEC growth *Naked Plasma Technique* – plasmid DNA increases VEGFR-3 expression *Viral Vectors/Adenovirus/Lymfactin –* adenovirus and associated gene vectors encode VEGF-C which regenerate lymphatic vessels *mRNA vectors (adeno associated)* – mRNA encodes VEGF-C which stimulates lymphatic growth (ii) HGF – Stimulation of LEC proliferation (iii) 9-cis RA – Works by activation of PI3K/AKT pathway to stimulate LEC proliferation (iv) ADSCs – increases LEC growth (v) hADSCs – mediate release of VEGF-C (vi) Recombinant human FGF2 – increases VEGF-C and VEGF-D release
Anti-inflammatory	(i) Ketoprofen (NSAID) – Inhibition of LTB4 AND 5LO (ii) Bestatin (NSAID) – Inhibition of LTA4H and LTB4 (iii) Fingolimod – Inhibition of activated CD4+ T cells (iv) Neutralising Antibodies – Inhibits Th2 inflammatory cytokines and IL4 (v) Doxycycline – Inhibits Th2 differentiation and monocyte recruitment (vi) Tacrolimus – Inhibition of IL2 mediated CD4+ T cells
Anti-fibrotic	(i) Anti-TGF-β1 - Inhibits TGF-β1 which disrupts myofibroblast maturation (ii) Pirfenidone - Inhibits TGF-β1 (iii) Captopril – ACE Inhibitors halt intracellular TGF-β1 pathways

An emerging treatment for established BCRL is the implantable nanofibrillar scaffold device (BioBridge; Fibralign Corporation, Union City, CA, USA), which has been investigated principally as an adjunct to procedures such as LVA and vLNT. Retrospective cohort data have shown that BioBridge combined with LVA or vLNT was superior to those interventions alone with regard to oedema reduction, new lymphatic formation, and dermal backflow reduction^132^. Animal models have demonstrated promising results using this technology as a preventative measure at the time of initial lymph node surgery^133^. Clinical trials of Lymfactin^®^, an adenovirus-based gene therapy and pro-lymphangiogenic growth factor vector that induces vascular endothelial growth factor C (VEGF-C) expression, have not shown any significant improvement in primary outcomes of lymphoedema severity compared with vLNT alone, although there was improvement in some secondary outcomes and the treatment was well tolerated^134^. Both these novel therapies will continue to be areas of active research in the coming years.

There remains a challenge when it comes to standardized and validated outcome assessments. Arguably, any outcome needs to include three aspects: function of the lymphatic system (that is lymphoscintigraphy with transport index, or ICG with MDA scale); volume/content assessment (that is perometry, circumferential assessment, MRL, or BIA); and patient-reported outcomes and symptoms including infections, range of motion, and quality of life. Currently, a combination of outcome measures is used in clinical studies, resulting in heterogeneity in the literature and limiting the possibility for comparison of outcomes. Going forward, a unified approach to outcomes among major centres using validated assessment tools is required.

#### Summary

BCRL is an extremely complex and challenging consequence of breast cancer treatment, negatively impacting patient quality of life and function, and requires lifelong management. Its development is multifactorial, and all physicians managing breast cancer patients must recognize the contribution of local, regional, and systemic therapies to the development of BCRL. Novel surgical methods for ILR have been shown to be effective and will have an increasing role in BCRL prevention. Improved methods of surveillance with early, longitudinal, validated screening programmes are required to optimize treatment and patient outcomes.

### Part 5—How to teach axillary surgery for the future breast surgeon?

Isabel T. Rubio and Pedro F. Gouveia

#### Introduction

Breast cancer treatment has evolved substantially in the past 30 years resulting in an improvement in both survival rates and the quality of life of breast cancer patients. In the past few years, multiple prospective randomized trials have altered contemporary management of the axilla in breast cancer surgery, with SLN surgery replacing ALND in the adjuvant and neoadjuvant setting, including the TAD technique^8,12,62,135–138^. Three decades ago, the training of residents in breast surgery seemed enough for practicing, because the breast surgery done at that time was reasonably learned during the residency programmes. Nowadays, the complexity of breast cancer surgery including oncoplastic procedures, such as nipple- and skin-sparing mastectomies, as well as complex axillary management algorithms in primary surgery and neoadjuvant settings have shown the need for an advanced level of training^139,140^. A recent survey of breast surgeons across Europe^141^ revealed wide variation in training and specialization in breast surgery with 12% of surgeons treating fewer than 25 cases per year and only a third of surgeons self-identifying as breast specialists. Whilst 77% of surgeons have had some focused oncoplastic training, 8% have had none at all. In addition, as ALND rates are rapidly decreasing over time, there will be an impact on the development of the operative skills of trainees, as well as the consequent effect on complications and morbidity. A decrease in rates of ALND has been shown by Rosenberger *et al*.^142^ from 32% in 2004 to 16% in 2014. This reduction was independent of hospital volume, facility type, timing of chemotherapy, or type of primary breast operation. Over the same interval, there was a clear reduction in the operative training of trainees. The absolute number of cases performed was low, with the average chief resident performing only a single major ALND case in their chief year. Recent evaluation of Society of Surgical Oncology/American Society of Breast Surgeons Breast Surgical Oncology Fellows demonstrated that, overall, most fellows meet the minimum case requirement of ten ALNDs in their fellowship year. However, there has been a decrease in the mean number of ALNDs/fellow/year from 23.2 in 2016 to 19.0 in 2024 (*P* < 0.001). Similarly, a survey sent to UK breast surgery trainees^143^ with questions related to level of training and experience in performing ALNDs showed that the majority (25.26%) of those who responded reported performing only one to ten ALNDs per year. Most had also performed only one to ten ALNDs during their career, resulting in breast surgery trainees not getting adequate training in ALND. This also impacts on confidence to perform the procedure independently. Even though 70% of breast cancer surgery worldwide is performed by general surgeons, it is becoming clear that residents have less experience in more complex cases, particularly axillary management and oncoplastic techniques, which has the potential to negatively affect patient care^144^. With the increased complexity of breast surgery procedures, skill acquisition from only the operating room is no longer enough for training. A modern breast surgeon uses a wide range of surgical techniques and an increasingly complex adjuvant and neoadjuvant decision-making algorithms, showing the need for additional training besides residency training. Moreover, both now and in the future, ALND will be more difficult and demanding as it will be used in patients with high axillary disease burden after neoadjuvant treatments and/or regional recurrences.

In a similar way the European Breast Surgical Oncology Certification (BRESO) (https://breastsurgeoncertification.com) requires 20 axillary lymph node surgeries, including both full axillary dissections and SLN surgeries^145^. To be considered a specialist breast surgeon and a member of the core team, each surgeon of the Breast Centre must personally carry out the primary surgery on a minimum of at least 50 newly diagnosed cancers per annum^146^. Surgeon specialization has also been linked to improved outcomes, and procedure-specific volume may also play a substantial role in outcomes. There is evidence that surgeon specialization is associated with both enhanced survival outcomes (up to 8% improvement at 10 years) and higher levels of patient satisfaction. Patients of ‘breast-focused’ surgeons have been shown to have better satisfaction with their care regardless of the sex of their surgeon, length of time in practice, or association with a cancer centre^147,148^. In the study by Kingsmore *et al*.^149^, survival from breast cancer was 20% worse when the patients were ‘not’ treated by a breast ‘specialist’. Kingsmore *et al*.^149^ concluded that this result was at least in part due to inadequate local treatment, resulting in a higher rate of local recurrence in the breast and axilla.

There is also evidence that caseload correlates positively with outcomes, again supporting specialization at both surgeon level and hospital level. Skill acquisition requires adequate training, certification, and ongoing re-accreditation to ensure practitioners keep up to date with the rapid pace of change in this discipline^140^.

The traditional and most effective way to learn surgical skills has been through the performance of operations under supervision; however, there is an increasing need to develop methods of teaching technical skills outside of the operating room.

#### Simulation-based training

Simulation can be a standardized and safe method for training and assessing surgeons^150^. The past three decades have seen a rising interest in the use of simulation for the purposes of training doctors, quality of care, and patient safety. The use of simulation became of heightened importance during the COVID-19 pandemic. It may be one potential method of increasing training opportunities whilst providing an opportunity for improving skills in a safe and controlled environment^143^. The Association of Surgeons in Training (ASiT) organized a Breast Skills Study Day using simulator models for three index procedures (wide local excision, wire-localized excision, and mastectomy/ALND), with consultants and registrars on hand, for trainees with limited breast experience^151^. One hundred percent of the participants ‘strongly agreed’ or agreed’ that the simulators provided good haptic feedback. In breast surgery, specifically, simulation has been described previously for SLN surgery^152^. More recently, an oncoplastic breast simulator has been successfully validated for assessment of technical skills in oncoplastic surgery^153^. However, other studies have shown that simulators are limited by their lack of realism, loss of haptic feedback, and limited ability to simulate an open procedure^154^.

#### Cadaver labs

Dissection of human donor tissues has always been an integral part of general surgery training^155^. Students find cadaver dissection to be ‘more effective than most educational interventions’ in teaching anatomy^156^. In addition to teaching anatomy, cadaver courses have also been found to be valuable in technical skill training. These laboratories provide a simulation of the real clinical operations. Studies have suggested that these skill laboratories supplement a surgeon’s learning experience^155^. Dissection-based training on human donors allows trainees to understand the anatomy and perform hands-on techniques that improve their skills. It can also contribute to gaining confidence for the next step when doing it independently. Different axillary surgeries can be performed and there are already models to teach SLN surgery by placing radioactive discs in the lymph nodes of donor tissue. In this model, the training also includes identification of axillary anatomy to take full advantage of a cadaver lab and have trainees become familiar with the anatomy of the axilla for axillary surgery^152^. Residents find training through the dissection of donor tissues to be helpful in learning anatomy, improving their confidence in performing these operations, and acquiring skills that are transferable to the operating room^157^. There are, however, limitations regarding human donors, such as cost, and variability in sex and breast size. Despite these limitations, cadaver training models remain the ‘gold standard’ for anatomic reality that cannot be obtained with simulators. As breast cancer surgery continues to evolve, cadaveric skill laboratory training is starting to be implemented for training in robotic mastectomies, with cadavers being considered one of the best options for robotic training. Due to limited resources, porcine models may be used as alternatives^158^. In addition to cadaver training, online teaching as well as handbook development may be valuable tools that can facilitate the improvement of the skill training programme.

#### The metaverse—virtual reality (VR) and augmented reality (AR)

Teaching axillary surgery poses unique challenges, primarily due to small axillary incisions with limited exposure. This restricted visual and physical access makes it difficult for surgical trainees to fully visualize anatomy and internal structures, such as lymph nodes, blood vessels, and nerves, which are critical for an accurate surgical technique. As the role of surgical axillary staging in breast cancer continues to evolve (from ALND to the emergence of SLN surgery and targeted techniques)^8,135,138^, this confined workspace demands advanced skills in instrument handling and three-dimensional (3D) spatial awareness. Additionally, the number of trainees allowed to be physically present in an operating room is inherently restricted^159^. With increasing demands on surgical facilities, using operating rooms for educational purposes has become increasingly difficult^159^. To overcome these barriers, the metaverse presents a groundbreaking opportunity for breast surgery education by leveraging a 3D digital environment accessible via VR and AR headsets (head-mounted displays)^160^. This technology allows users to create avatars and engage in immersive, interactive learning experiences through the internet^161^, enabling realistic surgical simulations and collaborative training beyond the constraints of physical spaces. Innovative technologies like VR and AR can provide enhanced visualization and allow trainees to practice in simulated risk-free environments, improving their confidence and competence before performing on real patients^162^. But VR and AR are quite different technologies with different educational applications. VR is a computer-generated environment that simulates reality within an immersive 3D setting^163^. This so-called virtual world is easily accessed through a VR headset and offers a transformative potential to revolutionize surgical training, unlocking new and innovative possibilities for skill development and education. Several studies have emphasized the potential role of VR in surgical education, demonstrating the effectiveness of VR in improving surgical competency, reducing the learning curve for specific surgical procedures, and allowing for data-driven insights, with quantifiable metrics on performance, such as precision and completion times^164^.

One of the most successful and practical uses in medical education is teaching anatomy with VR^165^, which improves anatomical knowledge, and offers a detailed 3D and immersive visualization of critical structures, like vessels, nerves, lymph nodes, and muscles, along with anatomical variations, that is crucial in axillary surgery. Although the promise of these new immersive technologies with regard to surgical education is growing, there is a lack of simulation apps involving axillary surgery. Continued research and innovation is needed to further develop VR-simulated scenarios, which ultimately should include effective haptic feedback integration, to enable learning tactile sensation during tissue handling^166^. They should also include handling of ultrasound and localization probes, as well as instrument resistance and lymph node palpation. But while specific VR tools are still not available for axillary surgery, AR-assisted remote telementoring can overcome some current limitations with a solution that immediately improves training accessibility and effectiveness. A recent study explored the feasibility of surgical remote telementoring using AR for breast surgery education^167^. Two surgeons, located remotely from each other, connected in real time during conservative breast cancer surgery. The remote surgeon provided visual guidance and oversight through an augmented view. Surgical incision placement was projected as a virtual image onto the trainee’s AR headset, ensuring precision without obscuring visibility. While this technique could benefit axillary surgery training, challenges include limited light visibility in small axillary incisions and reliance on supervisor availability, which may hinder scalability.

#### Artificial intelligence (AI)

AI emerged as a novel concept that can potentially reinvent the way surgery is practiced and taught. The concept has been recently defined as ‘An intelligent system/program that acts to fulfill or support the fulfillment of educational tasks traditionally performed exclusively by Surgical Educators, through making decisions in a manner similar to educators and providing customized adaptation, including performance assessment and feedback, to surgical trainees’^168^. Structural changes in surgical training workflows are needed to implement this radically new technology that will ultimately impact every surgeon, regardless of age or experience^169^. The need for a lifelong learning attitude in surgery will be redefined by the introduction of virtual AI agents as educators and supervisors. Specifically, large language models (LLMs) like GPT or MedGemini have been designed to analyse extensive medical data and natural language to support clinical decision-making, medical education, and administrative workflows^170,171^. For breast surgery training as a whole, LLMs can simulate case-based discussions, guide decision-making processes, and provide evidence-based recommendations by summarizing surgical guidelines. They can also offer real-time assistance during VR- or AR-based training, video-based evaluation and be used to generate performance metrics^169,170^. The concept of learning by repetition in surgical training will be enhanced with feedback powered by virtual agents, including performance metrics. But this new emergent path should also include further discussions and regulation on how to address biases, data privacy, and ethical concerns to ensure safe and effective use^172^.

#### Technique mastery

Other teaching options for breast cancer surgery (for example SLN surgery, ALND, and TAD) include video tutorials, live demonstrations, and supervised hands-on workshops. The use of courses and workshops (hands-on and video tutorials) to teach technical skills is not new to surgery. Such courses are, in fact, a mainstay of continuing education programmes for practicing surgeons. Extensive efforts have been employed to refine these training courses to maximize exposure for participants. Examples include: https://www.essoweb.org/courses/, https://surgonc.org/fellows/breast-fellows-webinars, https://associationofbreastsurgery.org.uk/courses-events/abs-courses-events, and https://www.eso.net/en/what%2dwe%2ddo/1-5018-1-.

#### Summary

The operating room has been the vehicle for trainees to acquire the surgical skills to perform surgery, although in breast cancer surgery this strategy may no longer be enough and optimal. Rather than seeing this trend as an adverse impact on surgical education and intraoperative skill development, it should be looked at as an opportunity to review how the next generations of breast cancer surgeons need to be trained. Although some experience with breast surgery is obtained across the residency training interval, the current complexity in breast cancer surgery (with less volume techniques such as ALND, or complex management of axillary surgery, and techniques for NSM and oncoplastic breast cancer surgery) demands the skill of specialized breast surgeons. Besides the acquisition of skills in operating rooms, the use of simulators, cadavers, hands-on courses, VR and AR teaching procedures, and LLMs will provide future breast cancer surgeons with skills necessary to standardize operative procedures. This will provide high-quality care to breast cancer patients, that will have an impact on oncological outcomes, as well as quality of life.

## Data Availability

No original data available to share.
